# Cognitive brain lateralization through neurovascular coupling in healthy subjects: A statistical complexity analysis

**DOI:** 10.14814/phy2.70492

**Published:** 2025-08-07

**Authors:** Héctor Rojas‐Pescio, Lucy Beishon, Ronney B. Panerai, Max Chacón

**Affiliations:** ^1^ Departamento de Ingeniería Informática Universidad de Santiago de Chile Santiago Chile; ^2^ Department of Cardiovascular Sciences University of Leicester Leicester UK; ^3^ NIHR Leicester Biomedical Research Centre University of Leicester Leicester UK

**Keywords:** brain lateralization, cognitive examination, dispersion entropy, neurovascular coupling, statistical complexity

## Abstract

Human sensory, cognitive, and motor processes often result in asymmetric cerebral hemisphere activation, observable through neurovascular coupling (NVC). Brain lateralization enables simultaneous performance of distinct functions, enhancing cognitive capacity. This study examined cognitive lateralization through NVC responses to the Addenbrooke's Cognitive Examination‐III (ACE‐III) assessment, using entropy‐based methods and statistical complexity measures (SCM). We tested whether applying *dispersion entropy* (DE) to cerebral blood velocity (CBv), critical closing pressure (CrCP), and resistance area‐product (RAP) signals could identify significant hemispheric differences during cognitive tasks. Statistical analysis revealed SCM effectively detected lateralization (best *p*‐value = 0.001), whereas entropy alone did not differentiate hemisphere activity. Furthermore, cognitive stimulation (attention, fluency, language, memory, and visuospatial tasks) generally produced lower SCM values compared to baseline, predominantly in the dominant hemisphere. These findings indicate that NVC exhibits distinct complexity patterns based on hemisphere dominance and cognitive domain stimulated. Additionally, comparison with prior ACE‐III analyses, using population‐normalized mean peak change, reinforces that advanced biomedical‐oriented information theory methods, such as DE and SCM, offer valuable insights into cerebral lateralization mechanisms and NVC responses during cognitive stimulation.

## 1 INTRODUCTION

Since the 17th century, evolutionary advantages associated with hemispheric lateralization and their associated cognitive functions have been studied (Broca, XVII; Wernicke, XIX; Cunningham, XIX; Henschen, XIX). According to recent research, brain lateralization begins ontogenetically in many species with asymmetrical gene expression patterns within the nodal cascade, laying the basis for more complex interactions (influenced by genetic, environmental, and epigenetic factors) that impact at various stages of ontogeny, resulting in neural network asymmetries across species (Güntürkün & Ocklenburg, 2017; Letzner et al., 2017; Malatesta & Tommasi, 2023). As a result, depending on the task requirements, feedforward or feedback projection loops in either the left or right hemispheres dominate neuronal activity. However, other brain characteristics such as asymmetries in commissural transmission can affect lateralized processes in both hemispheres (Güntürkün & Ocklenburg, 2017). At present, various *psychophysiological measurements* (measure of the physiological responses in the context of psychological processes and experiences activated through cognitive stimulation), such as cortical electric activity and cerebral hemodynamic mechanisms, are being used to understand different brain hemisphere functions, for example, organization of the cerebral cortex in relation to different aspects of cognitive function (attention, fluency, language, memory, motor control, visuospatial, and, among others) and brain hemisphere specialization.

Of the above, neurovascular coupling (NVC) is responsible for delivering nutrients and oxygen, as well as removing metabolic waste such as carbon dioxide, during neuronal activation. Raised neuronal activity works via NVC to increase cerebral blood flow (CBF) to meet the brain's metabolic demands (Hosford & Gourine, 2019; Iadecola, 2017). NVC has been shown to change in both healthy and diseased states, and it can be measured indirectly with various imaging modalities such as magnetic resonance imaging (MRI), near‐infrared spectroscopy (NIFS), positron emission tomography (PET), and transcranial Doppler ultrasonography (TCD). Different protocols have been used to measure NVC during cognitive stimulation, which can vary from visual to motor (passive and active arm movement) and cognitive (task activation) (Beishon et al., 2021; Leacy et al., 2022; Rosengarten et al., 2006; Salinet et al., 2018; Stroobant & Vingerhoets, 2000).

Aaslid (1987) was the first to demonstrate TCD‐measured changes in cerebral blood velocity (CBv) in the posterior cerebral artery (PCA) with visual stimulation. Also, changes in CBv subcomponents such as the critical closing pressure (CrCP), which represents the pressure inside a blood vessel below which the vessel will collapse, and resistance area product (RAP), which represents the inverse slope of the instantaneous beat‐by‐beat relationship between CBv and blood pressure during NVC (Evans et al., 1988), have been analyzed during cognitive stimulation (Beishon, Williams, Robinson, et al., 2018). Subsequently, many studies have found similar results using neurocognitive paradigms in the middle cerebral artery (MCA), for example, with verbal and motor tasks (Azevedo et al., 2010; Salinet et al., 2018). This response causes a 10%–20% rise in CBF in the PCA and a 5%–8% increase in the MCA in healthy individuals (Girouard & Iadecola, 2006). Other research has also shown hemispheric lateralization of CBv delivery by using either right‐handed or verbal activities, or left‐handed motor paradigms to preferentially engage the left and right MCAs, respectively (Moody et al., 2005). In contrast, in previous studies by Beishon, Williams, Panerai, et al. (2018), Beishon, Williams, Robinson, et al. (2018), there was no lateralization of the NVC in healthy participants when using the Addenbrooke's Cognitive Examination‐III (ACE‐III), analyzed by the *population‐normalized mean peak change in CBv*.

However, to understand the nonlinear dynamics of cognitive function, complexity‐driven methods provide powerful tools for understanding the brain's dynamic interactions (Telesford et al., 2011). Alternative approaches that use measures of entropy and statistical complexity have been researched to a lesser extent in psychophysiological signal analysis to study cognitive effort and brain lateralization. For example, Zhang et al. (2021) analyzed functional near‐infrared spectroscopy signals in children with autistic spectrum disorder using *multiscale entropy* and found significantly increased complexity in the resting state in the left temporal lobe. Furthermore, Wang et al. (2023) proposed the *multiscale lateralized brain entropy* method to assess mild cognitive impairment and Alzheimer's progression.

Therefore, the purpose of this study was to determine brain lateralization of NVC responses to ACE‐III‐induced cognitive stimulation utilizing information entropy‐based approaches and statistical complexity measures (SCM) based on the distance between probabilities, known as disequilibrium. We test the hypothesis that by applying the appropriate entropy family to brain hemodynamic signals, CBv, CrCP, and RAP, we can distinguish between dominant and non‐dominant brain hemodynamic signals during mental activation generated by ACE‐III cognitive tasks better than through *population‐normalized mean peak change analysis*.

## 2 MATERIALS AND METHODS

### 2.1 Subjects and measurement

Data were collected previously at the Cerebral Hemodynamics in Aging and Stroke Medicine (CHiASM) laboratory at the University of Leicester. All participants provided informed, written consent, and the study had ethical approval from the University of Leicester (ref: 5355vjh12cardiovascularsciences). All procedures complied with the principles of the Declaration of Helsinki (1975), including subsequent revisions in 2008 and 2024. Written informed consent was obtained from all participants prior to their inclusion in the study.

In brief, 40 healthy participants (median age 31 years, 68% female, 93% right‐hand dominant) completed all 20 tasks from the Addenbrooke Cognitive Evaluation (ACE‐III), with continuous monitoring of bilateral CBv in the middle cerebral arteries (MCA) using TCD, heart rate (3‐lead electrocardiogram ECG), end‐tidal CO_2_ (EtCO_2_, nasal capnography), and blood pressure (BP, Finometer). The ACE‐III is a brief cognitive screening tool used in routine clinical practice to assess performance in five cognitive domains: attention, verbal fluency, language, memory, and visuospatial (Hsieh et al., 2013). For this study, the ACE‐III was conducted in the same order that it is undertaken clinically, and the test was divided into three recordings: A section (four attention, two verbal fluency, and three memory tasks), B section (six language tasks), C section (four visuospatial and one memory task) (Hsieh et al., 2013) as detailed in Table 1. Data were stored in the PHYSIDAS acquisition system for offline analysis. All data were inspected visually, and large non‐physiological spikes were removed using linear interpolation. CBv recordings were passed through a median filter to remove smaller non‐physiological noise, and all recordings were low‐pass filtered with a zero‐phase Butterworth filter with a cutoff frequency of 20 Hz. The R‐R interval was derived from the 3‐lead ECG to determine heart rate (HR) and allow calculation of mean beat‐to‐beat values of CBv, EtCO_2_, and BP. A detailed description of the methods used for data collection and signal pre‐processing can be found in previous publications Beishon et al. (2017), Beishon, Williams, Panerai, et al. (2018). An analysis of variance (ANOVA) power analysis was performed to identify the optimal number of observations for each section analysis for baseline and paradigms, with a significance threshold of 0.05 and a power of 0.99. Sections A, B, and C each required 32, 26, and 28 observations, respectively. To calculate the CrCP and RAP values for each cardiac cycle, the instantaneous and linear relationship between CBv (in the ordinate) and BP (in the abscissa) of a Cartesian plane is used. The CrCP represents the intercept with the BP horizontal axis, where the CBv is equal to zero, and the slope of the regression line represents the inverse of the RAP (Evans et al., 1988).

**TABLE 1:** ACE‐III tasks used to elicit CBv responses, task durations, and the best post hoc test obtained *p*‐values when analyzing CBv signals' dispersion entropy (DE) statistical complexity measures (SCM) distributions^a^ and population‐normalized mean peak change (PMPC) analysis^b^ for each paradigm dominant and non‐dominant hemispheres.

Task	Domain	Detail	Task duration, s	*p‐*Values SCM^a^	*p‐*Values PMPC^b^
*A section*					
A1	Attention	Orientation to time (day/date/month/year/season)	15.7 (5.4)	**0.014**	0.990
A2	Attention	Orientation to space (floor/building/town/county/country)	14.8 (2.5)	**0.020**	0.540
A3	Attention	Repeat and remember three words (lemon/key/ball)	14.5 (1.9)	**0.014**	1.000
A4	Attention	Subtract serial sevens from 100	27.5 (11.5)	**0.014**	0.950
A5	Memory	Recall the three words learnt earlier (A3: lemon/key/ball)	7.0 (1.8)	**0.005**	0.640
A6	Fluency	Naming words beginning with “P” in 1 min	96.8 (8.9)	**0.010**	**0.003**
A7	Fluency	Naming animals in 1 min	73.8 (7.3)	0.072	1.000
A8	Memory	Learn and remember a name and address	60.3 (10.0)	**0.047**	0.770
A9	Memory	Names of current and previous UK prime minister and US president	25.3 (4.7)	0.261	0.610
*B section*					
B1	Language	Following verbal instructions	32.2 (4.5)	**0.007**	0.150
B2	Language	Writing two sentences	58.9 (15.8)	**0.049**	0.014
B3	Language	Repeating words and phrases aloud	27.4 (3.9)	0.109	1.000
B4	Language	Naming objects	26.0 (11.4)	**0.007**	0.900
B5	Language	Linking objects with statements	25.7 (7.0)	**0.002**	**<0.005**
B6	Language	Reading words aloud	11.0 (2.7)	0.160	1.000
*C section*					
C1	Visuospatial	Drawing an infinity diagram and three‐dimensional cube	41.8 (13.7)	0.238	1.000
C2	Visuospatial	Drawing a clock face and correctly positioning the hands to a given time	41.57 (10.83)	0.065	0.980
C3	Visuospatial	Counting number of dots	23.4 (6.8)	0.151	0.940
C4	Visuospatial	Recognizing obscured words	8.6 (2.7)	**0.037**	0.990
C5	Memory	Recalling the previously learnt name and address (A8)	20.0 (13.3)	0.085	0.990

*Note*: Values are means (SD) for duration of Addenbrooke's Cognitive Examination (ACE‐III) tasks used to elicit cerebral blood velocity (CBv) responses. All statistical tests had a significance level of 0.05, *p*‐values lower than the significance level are highlighted in bold font.

### 2.2 Methods

#### 2.2.1 Statistical complexity

Information theory has been used for the study of dynamic systems for decades (Kolmogorov, 1958; Sinai, 1959). Information about the dynamic phenomenon underlying a signal can be quantified by examining stochastic processes based on information entropy. Entropy corresponds to a measure of the degree of order of a signal, and it can be considered as a measure of the uncertainty associated with phenomenological processes. The measure of the information *H* contained in any arbitrary probability distribution $P=\left\{\mathrm{pi}:i=1,\ldots ,M\;\right\}$, with M degrees of freedom is expressed as:

(1) $$H=S\left[P\right]=-\sum_{i=1}^{M}p_{i}\ln\left(p_{i}\right)$$

When $S\left[P\right]=S_{\min}=0$, the possible outcomes of S can be predicted with 100% certainty, and their probabilities are given by $p_{i}$. The knowledge of the underlying process described by the probability distribution in this case is maximum. In contrast, for a uniform distribution ($P_{e}=\left\{\frac{1}{M},\forall_{i}=1,\ldots ,M\right\}$), the uncertainty is $S\left[P_{e}\right]=S_{\max}=\ln\;M$, with minimum knowledge of the process (Shannon, 1948).

In this study, the *dispersion entropy* (DE) introduced by Rostaghi and Azami (2016) was utilized. This method corresponds to an irregularity indicator of the same name, since dispersion in statistics refers to a manner of identifying how spread out a set of data is, considering differences between amplitude values and the mean (or median) value of amplitudes, and as well as omitting relevant amplitude information from the time series. DE has been utilized in the study of dynamics of biomedical signals such as in Azami et al. (2017) and Diykh et al. (2023) with an acceptable time series characterization performance.

As described by Rostaghi and Azami (2016), when observing a given univariate signal $x=\left\{x_{1},x_{2},\ldots ,x_{N}\right\}$ with length N, the DE algorithm includes 4 main steps as follows:

1. The signal x is classified into c classes using linear and nonlinear mapping techniques (examples are included in Appendix A1).
2. Using an embedding dimension m and time delay τ, time series $u_{i}^{m,c}$ are made were $i=1,2,\ldots ,N-\left(m-1\right)\tau$. Each time series $u_{i}^{m,c}$ is mapped to a dispersion pattern $\pi_{v_{0}v_{1}\ldots v_{m-1}}$. Because the signal $u_{i}^{m,c}$ has m elements and each obtains an integer value in the range 1 to c, the possible number of dispersion patterns assigned to each vector $u_{i}^{m,c}$ is equal to $c^{m}$.
3. For each of $c^{m}$ potential dispersion patterns $\pi_{v_{0}\ldots v_{m-1}}$, relative frequency is calculated.
4. Finally, DE is computed based on the Shannon's definition of information entropy as shown in Equation (2):

(2) $$H_{\mathrm{DE}}\left(x,m,c,\tau \right)=-\sum_{i=1}^{N}p\left(\pi_{v_{0}\ldots v_{m-1}}\right)\ln\left(p\left(\pi_{v_{0}\ldots v_{m-1}}\right)\right)$$

Further details of DE equations can be found in Rostaghi and Azami (2016) and Azami and Escudero (2018). Also, a numerical example is included in Appendix A2.

Entropy, as a measure of uncertainty in probabilistic processes, frequently falls short of representing the deep interactions that exist inside complex systems such as biological ones (Zanin et al., 2012). To compensate for this limitation, statistical complexity measures (SCM), such as the product of entropy (H) and disequilibrium (Q) in Equation (3), discount randomness and provide a more thorough perspective of system behavior.

(3) C=H.Q

Disequilibrium (Q) is a distance between probabilities that allows examining the complicated dynamics of complex systems. This distance measure can be applied to any probabilistic space (Kowalski et al., 2011). Disequilibrium and their distances' Jensen‐Shannon divergence and Wooters' distance calculations that are included in Appendix A3.

In this study, the entropy‐complexity causality plane, or HC plane, was also used to analyze the NVC mechanism by applying a linear classifier to discriminate between baseline, dominant, and non‐dominant CBv signals and its subcomponents (CrCP and RAP) and to associate entropy and SCM values with the NVC mechanism's response to cognitive effort.

#### 2.2.2 Data analysis

**FIGURE 1:**
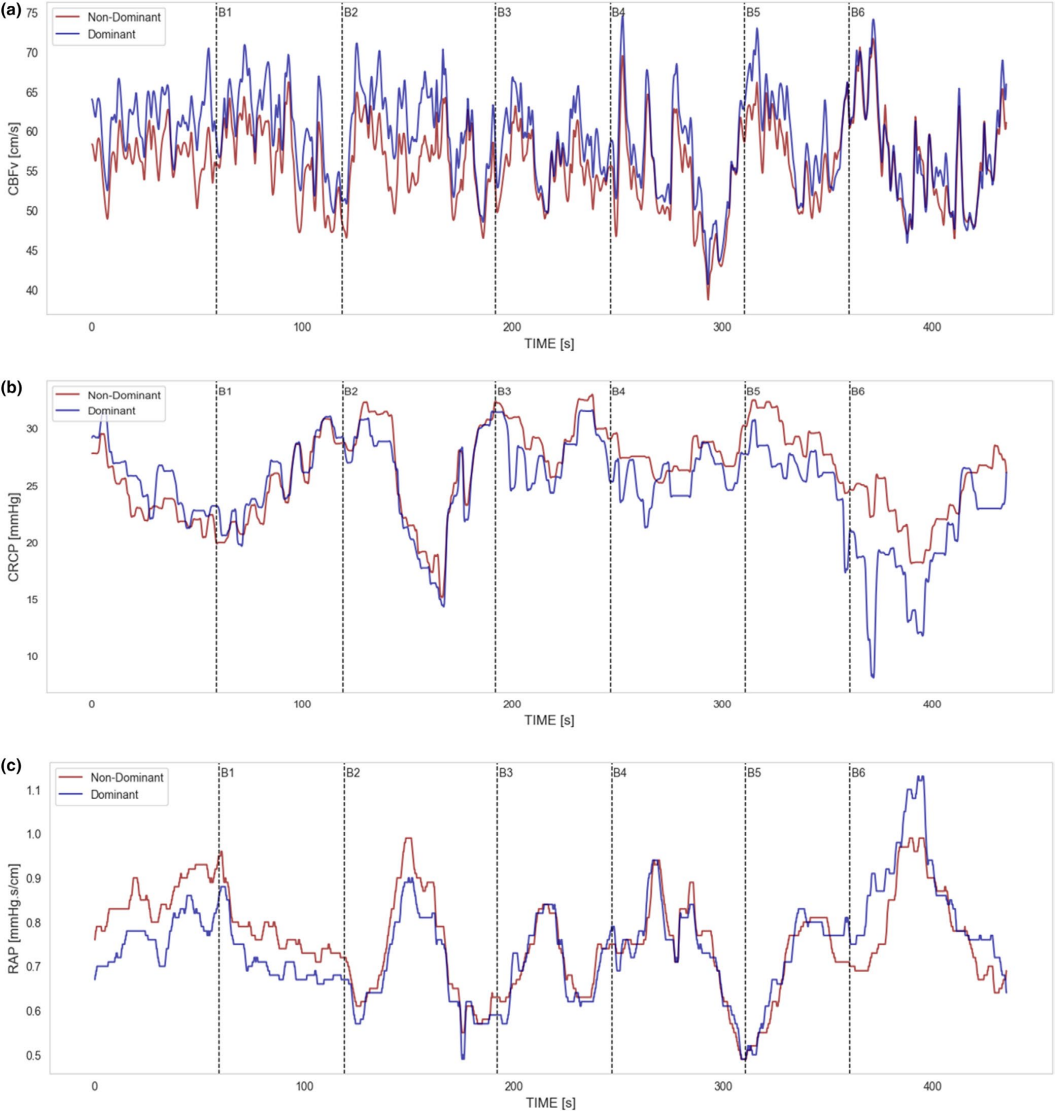
Dominant and non‐dominant hemodynamic signals, (a) Cerebral blood velocity (CBv), (b) Critical closing pressure (CrCP), and (c) Resistance area‐product (RAP) for subject #12 during the B section (language) cognitive tasks B1 to B6.

The Jupyter Notebook data science software with the Python 3.7 programming language was used to accomplish this study data analysis. To obtain dispersion entropy, the *EntropyHub* package by Flood and Grimm (2021) was installed. Then, entropy and statistical complexity measures (SCM) were obtained for each CBv, CrCP and RAP baseline, paradigms' dominant and non‐dominant signal subsamples as shown in Figure 1. Normalized dispersion entropy measures were calculated with embedding dimension parameter $m\in \left[2-4\right]$, number of symbols parameter $c\in \left[\;2-8\right]$, and the data‐to‐symbolic sequence transform algorithms *Linear, NCDF, Equal, and Finesort* were applied. SCM were calculated with a generalized version of the Jensen‐Shannon divergence and Wooters' distance (Kowalski et al., 2011), as shown in Appendix A3.

#### 2.2.3 Statistical analysis

To assess the normality of CBv, CrCP, and RAP signals, a Shapiro–Wilk normality test was first performed on the distributions of means obtained from subsamples of each subject's signals in Sections A (attention, fluency, and memory tasks), B (linguistic tasks), and C (visuospatial and memory tasks). Then, to determine statistically significant differences between dominant and non‐dominant hemisphere signals, mean distributions from subsamples corresponding to each subject paradigm were evaluated using a *t*‐test or Wilcoxon signed‐rank test, depending on normality test results.

Similarly, dispersion entropy and SCM distributions were used to evaluate differences between baselines and paradigms' dominant and non‐dominant signals in each section (A, B, and C). Repeated measures tests (analysis of variance ANOVA or Friedman chi square) were applied to dispersion entropy and SCM distributions based on earlier normality evaluation using the Shapiro–Wilk test. Later, post hoc analysis was used to compare baseline and non‐dominant signals, baseline and dominant signals, and non‐dominant and dominant signals using the paired Tukey HSD test or Nemenyi test. After correcting for repeated measures, results with a *p*‐value of ≤0.05 were considered statistically significant. Finally, normalized dispersion entropy and SCM distributions with distinctive post hoc outcomes were classified using a logistic regression model and visualized on the HC plane, including the decision boundary line, to infer about the NVC mechanism behavior. To address uncertainty due to the limited sample size (*n* = 40), we performed nonparametric bootstrapping with 1000 resamples to compute 95% confidence intervals (CI) for the ROC AUC and classification accuracy metrics obtained in the entropy‐complexity (HC) plane. For each bootstrap iteration, the classification model was refit using stratified sampling with replacement, and the corresponding ROC AUC and accuracy values were stored to generate empirical distributions and percentile‐based CIs. A Receiver Operating Characteristic Area Under the Curve (ROC AUC) score of 0.70 or higher was deemed acceptable.

## 3 RESULTS

Forty‐eight healthy volunteers were recruited, but eight were unable to participate due to poor window conditions (n=1), poor quality data (n=6), and equipment failure (n=1). The analyses comprised forty participants, the majority of whom were female (n=27), and right‐hand dominant (n=37), with a median age of 31 years [interquartile range (IQR): 22–52 years]. There were no severe neurological, behavioral, or medical comorbidities among the volunteers. Ninety percent of the volunteers were Caucasian, with the remaining ten percent of Southeast Asian or Far East Asian origin. The average ACE‐III score for all individuals was 98 (2.03), indicating that no‐one fell below the criterion for cognitive impairment (88) (Mioshi et al., 2006). Average task durations can be seen in Table 1. While the longest task was A6, with a mean duration of 96.75 (9.1) seconds, the shortest task was A3, with an average duration of 14.57 (1.89) seconds.

The Shapiro–Wilk normality test results showed that RAP signals had nonparametric distributions while CBv and CrCP signals had normal distributions.

The observed paired *t*‐test *p*‐values showed no significant differences in the majority of paradigms. Among the three hemodynamic signals and 20 cognitive tasks, only CrCP signals in paradigms A6, A7 (fluency), and B4 (linguistic) showed significant differences. Following that, the histograms of brain hemodynamic dominant and non‐dominant signals were viewed to examine hemispheric separability, the distributions showed an overlap greater than 90%, indicating a lack of hemispheric separability.

Then, using the previously stated data processing approach, DE measures and SCM were generated and evaluated for each paradigm signal using repeated measurements and post hoc testing. The obtained results showed that dominant and non‐dominant CBv signals could be differentiated through SCM values in most of the paradigms of sections A (attention, fluency, and memory tasks) and B (linguistic tasks) in comparison to DE measures, as shown in Table 1, where the best post hoc test achieved *p*‐values when evaluating CBv signals using DE statistical complexity measures distributions and population‐normalized mean peak change (PMPC) analysis. While PMPC analysis could discriminate two out of twenty tasks, SCM revealed substantial differences in 12 out of twenty tasks, with attention and language paradigms being the most distinguishable. In summary, in Section A, attention, 7 out of 9 verbal fluency, and memory paradigms (78%) could be distinguished [A1, A2, A3, A4, A5, A6 and A8]. In the case of Section B, 4 out of 6 language paradigms (67%) could be distinguished [B2, B4 and B5]; however, only 1 [C4] out of 5 visuospatial and memory paradigms could be distinguished in Section C.

This behavior could also be observed when assessing CrCP and RAP signals through DE and SCM, included in Appendix A4. In addition, the evaluation of dominant and non‐dominant hemisphere CrCP signals using dispersion entropy and SCM distributions (included in Appendix A4) could separate 8 out of 9 paradigms (88%) in Section A [A1 to A8] and 5 out of 6 paradigms (83%) in Section B [B2 to B5]. In Section C, 3 out of 5 paradigms (60%) could be differentiated [C1, C3 and C4]. Furthermore, the evaluation of RAP signals using the same entropic and complexity measurements (included in Appendix A4) confirmed the efficacy of the method. Similarly, for CrCP, in Section A, 5 out of 6 paradigms were distinguished [A2 to A9], but a lower performance was observed in Section B (language) with 2 out of 5 paradigms separated [B2 and B4]. Finally, better results were obtained in Section C paradigms, where 5 out of 6 dominant and non‐dominant hemisphere signals were identified [C1 to C4].

**FIGURE 2:**
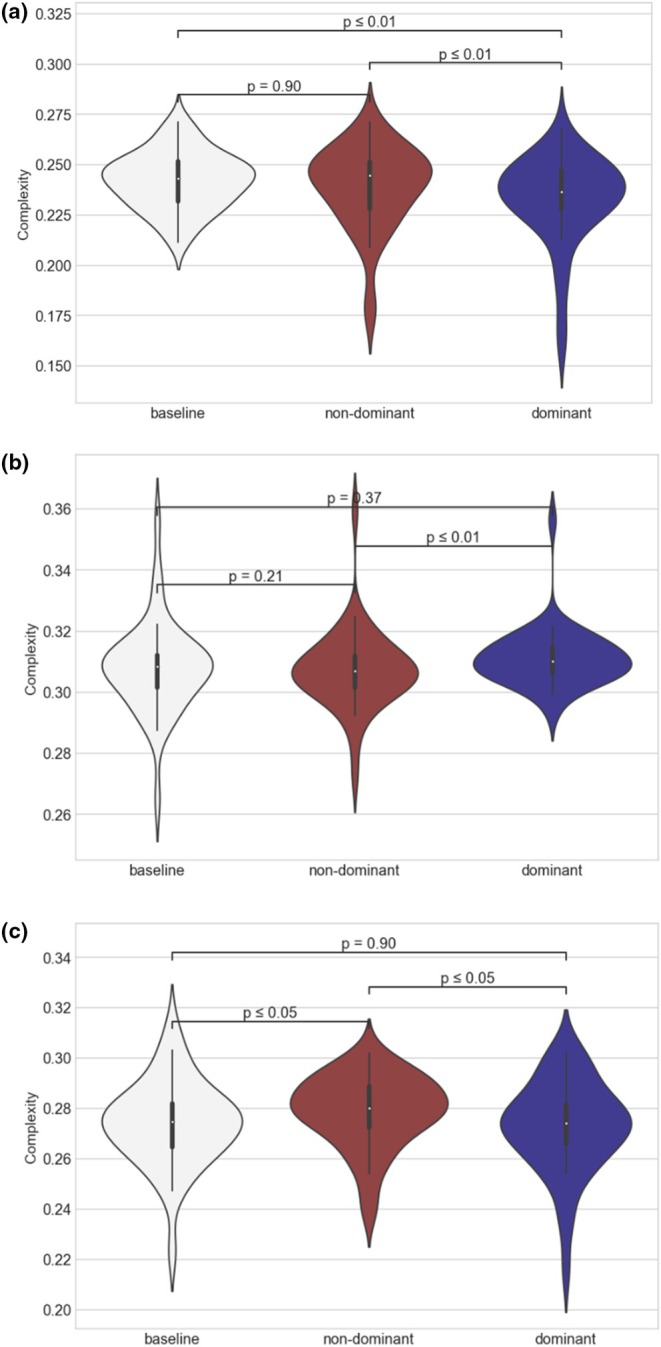
Violin plots with Friedman chi square *p*‐values, multiple SCM comparisons for baseline, non‐dominant, and dominant signals calculated from dispersion entropy, and SCM calculated with Wooters' distance. (a) Memory paradigm A5 (*Recall the three words learnt earlier*). (b) Language paradigm B4 (*naming objects*). (c) Visuospatial paradigm C4 (*recognizing obscured words*) Baseline in white, non‐dominant signals in gray, and dominant signals in dark gray.

When observing baseline, non‐dominant, and dominant CBv signals' SCM through violin plots (Figure 2), NVC responses showed lower median SCM values in dominant and non‐dominant hemispheres' signals in sections A (attention, verbal fluency and memory) and C (visuospatial and memory), relative to section B (language). Moreover, a Wilcoxon rank sum test applied over sections A, B, and C paradigms' SCM median distributions showed lower values in comparison to baseline in most sections' paradigms, but median complexity between dominant and non‐dominant hemispheres was conditional on cognitive tasks.

**FIGURE 3:**
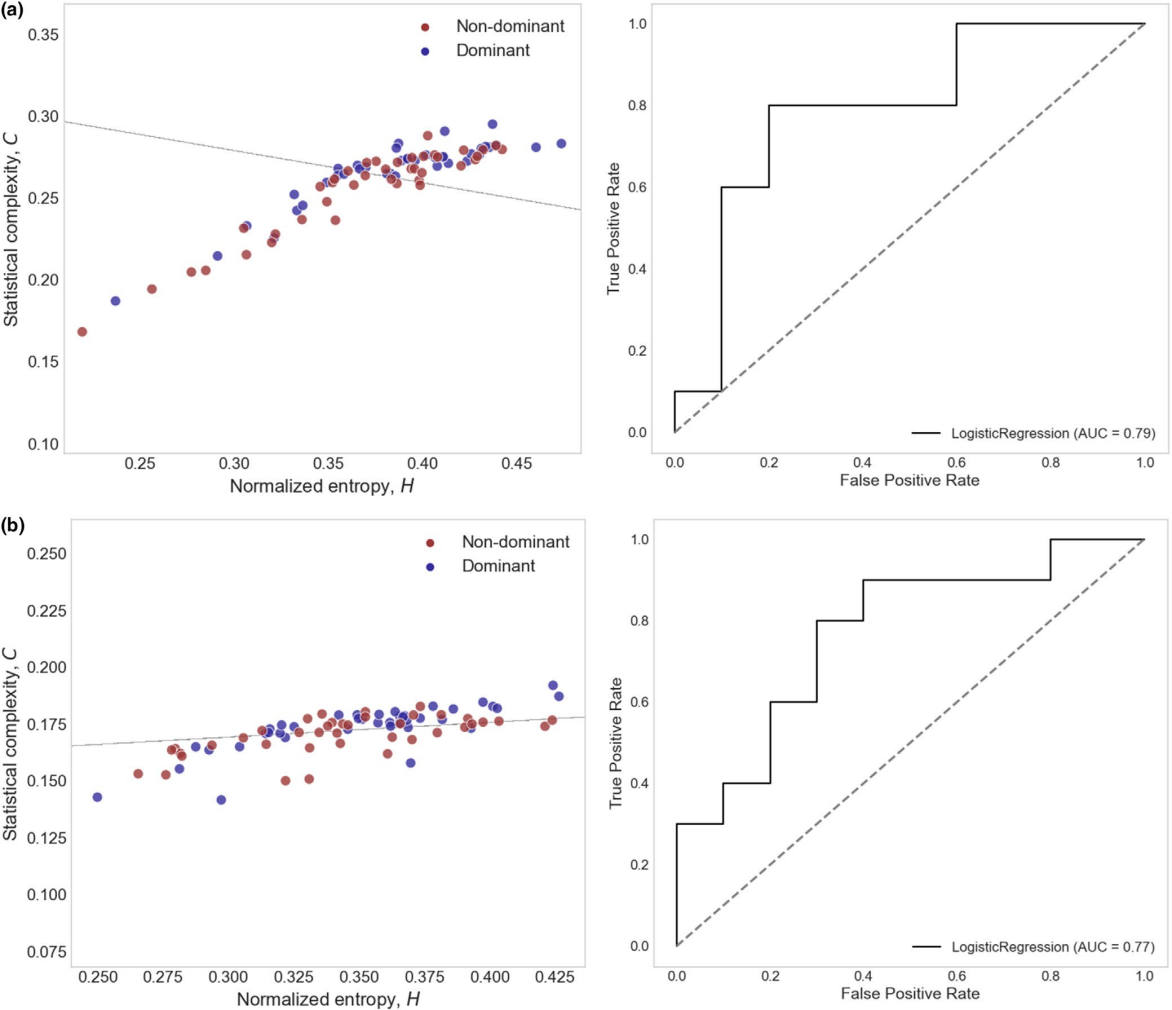
Logistic regression classification of the dominant and non‐dominant hemispheres in the HC plane. (a) CrCP signal from Section C, paradigm C1 (drawing an infinity diagram and a three‐dimensional cube), with an AUC of 0.79 (95% CI: [0.742–0.980]) and an accuracy of 0.75. (b) RAP signal from Section C, paradigm C4 (recognizing obscured words), with an AUC of 0.77 (95% CI: [0.714–1.000]) and an accuracy of 0.70.

Following that, logistic regression classification of CBv, CrCP, and RAP signals' entropy and SCM in the HC plane revealed that, when differentiating between resting baseline and paradigms from non‐dominant hemispheres and dominant hemispheres, acceptable ROC AUC scores (≥0.70) were achieved, with lower complexity values in comparison to baseline. Almost no satisfactory linear separations between the non‐dominant and dominant hemispheres were achieved. Appendix A5 summarizes the mean ROC AUC values derived in sections A, B, and C for CBv signal entropy and SCM classification. When examining paradigms from Sections A and C, only the CrCP and RAP signals demonstrated acceptable discriminative performance, as reflected by their ROC AUC scores. Specifically, the classification of the CrCP signal in Section C, paradigm C1 (*drawing an infinity diagram and a three‐dimensional cube*) included in Figure 3a, achieved an AUC of 0.79 (95% CI: [0.742–0.980]) and an overall accuracy of 0.75. Similarly, the RAP signal in Section C, paradigm C4 (*recognizing obscured words*) shown in Figure 3b, yielded an AUC of 0.77 (95% CI: [0.714–1.000]) and an accuracy of 0.70. The corresponding classifications in the HC plane, along with the ROC curves, are presented in Figure 3a,b.

## 4 DISCUSSION

The aim of this study was to determine brain lateralization through the NVC response to ACE‐III‐induced cognitive stimulation utilizing information entropy‐based approaches and statistical complexity measures (SCM) based on disequilibrium. Through our previous analyses, we observed that while all brain hemodynamic signals' histograms showed low separability, CBv and CrCP signal distributions were normal, and RAP signals exhibited non‐normal distributions.

The statistical analysis results in Table 1 demonstrated that brain lateralization could be distinguished by SCM, in contrast to the entropy measures, whose repeated measures and post hoc tests found almost no differences between brain hemisphere signals. Through Table 1 results, we could also validate our hypothesis that, by analyzing cerebral hemodynamic signals using SCM calculated with equilibrium‐based distance (in our study Shannon Jensen divergence and Wooters distance), we could distinguish better between dominant and non‐dominant signals during mental activation generated by ACE‐III cognitive tasks than population‐normalized mean peak change analysis (Beishon, Williams, Panerai, et al., 2018; Beishon, Williams, Robinson, et al., 2018).

CBv signals, according to our findings, might be utilized to assess hemisphere lateralization in attention, verbal fluency, memory, and language cognitive tasks, while visuospatial abilities could be studied to a lesser extent. CrCP signals, on the other hand, have shown that this signal can assess brain lateralization in almost all cognitive functions, notably language tasks. RAP signals can also evaluate attention, verbal fluency, and memory paradigms but are optimal in visuospatial paradigms. According to other studies that used the TCD technique to capture CBv signals to assess brain lateralization with the laterality index when performing language tasks (Woodhead et al., 2020), our results also revealed strong lateralization in word and phrase generation paradigms such as B2 (*writing two sentences*), B4 (*naming objects*), and B5 (*linking objects with statements*), as they were the most distinguishable through CBv signals' SCM analysis.

In order to examine NVC activity in response to cognitive effort using entropic and statistical complexity measures, we analyzed SCM distributions using median values since they lacked normality. As shown in Figure 2, baseline SCM measures influence (with no cognitive stimulus) had higher complexity in most of the cases. This is confirmed by the SCM median values of CBv signals, where lower median SCM values are highlighted per paradigm. While dominant hemisphere signals showed lower median values in language and visuospatial tasks, non‐dominant hemisphere signals showed lower median values in attention and verbal fluency tasks. Memory tasks showed lower complexity values in both hemispheres equally (paradigms A5, A8, A9, and C5).

When analyzing hemodynamic signals (CBv, CrCP, and RAP), logistic regression in the HC plane successfully distinguished between baseline and task‐related hemispheric signals. Dominant and non‐dominant hemisphere signals exhibited lower statistical complexity compared to baseline and were linearly separable with a low‐gradient boundary, indicating differentiation primarily along the complexity axis. While entropic measures remained similar across hemispheres, CrCP and RAP signals revealed stronger lateralization patterns than CBv. In particular, the CrCP signal during paradigm C1 (drawing an infinity diagram and a three‐dimensional cube) achieved an AUC of 0.79 (95% CI: [0.742–0.980]) and an accuracy of 0.75, while the RAP signal in paradigm C4 (recognizing obscured words) yielded an AUC of 0.77 (95% CI: [0.714–1.000]) and an accuracy of 0.70. These results reflect good discriminative capacity. CrCP and RAP produced better findings when examining brain lateralization because, unlike CBv, which is influenced by several parameters (e.g., vessel diameter and blood viscosity), CrCP and RAP directly represent pressure conditions in the cerebral vasculature (Panerai et al., 2021). Also, CrCP and RAP may provide insights into microvascular reactivity (Castro et al., 2012), which is essential for understanding NVC response.

It is also important to highlight, given that NVC responds to cognitive paradigms by producing changes in cerebral blood flow in neurofunction‐specific areas, for example, language is generally left‐hemisphere dominant, particularly for grammar, syntax, and vocabulary (Deppe et al. 2004), our findings showed that SCM can effectively distinguish between dominant and non‐dominant hemodynamic signal structure, as characterized by DE through the pattern extraction process. Among these findings, we may infer that the distribution entropy's data‐to‐symbolic sequence transformation from time series captured global changes in dominant and non‐dominant hemisphere CBv, CrCP, and RAP signals, and SCM correctly captured local changes produced by the dynamics of the NVC mechanism.

Among the clinical implications of this study, the favorable findings when analyzing attention, fluency, and memory paradigms through CBv and its subcomponents, can help reveal lateralization of attentional processes, which can contribute to a better understanding of illnesses such as *attention deficit hyperactivity disorder* (ADHD). Brain hemodynamic statistical complexity measures can also be used to assess lateralization of spatial cognition processing, especially in infants, which provides insights into spatial memory problems (Aslin et al., 2015). High precision in differentiating brain hemispheres during language paradigms using CrCP signal analysis can be used to predict language recovery potential after stroke.

While TCD is a valuable technique for studying brain hemodynamics, this study was cognizant of its limits and took caution with data analysis and result interpretation. CBv were captured in the MCA, which largely serves the frontal and parietal lobes. It is unable to distinguish between the anterior and posterior regions of these lobes, making it difficult to precisely locate neurofunction‐specific cortical areas. As a result, its limits in spatial resolution, indirect measurement of neuronal activity, and the influence of extracranial factors must be carefully considered in brain hemodynamics lateralization research.

Despite these promising findings, certain limitations must be acknowledged. The wide confidence intervals associated with the AUC values—particularly the upper bound of 1.000 for the RAP signal—suggest variability likely stemming from a limited sample size. Although the observed accuracy levels are acceptable for exploratory research, they may not meet the requirements for clinical or high‐stakes applications. Nonetheless, the results provide encouraging evidence that CrCP and RAP signals can predict hemispheric dominance above chance level. Further validation with larger, independent samples is needed to confirm their robustness and generalizability. Future studies should also explore the inclusion of additional features or ensemble approaches to improve classification stability. Lastly, consistent with prior statistical analyses, entropy estimates showed minimal hemispheric differences, and no generalizable linear separability was found across participants in the HC plane. An average AUC of 0.504 across all sections reinforces that hemispheric differences during ACE‐III stimulation are better captured through statistical complexity than entropy.

### 4.1 Further work

Future research should assess brain lateralization by examining hemodynamic signals recorded with TCD in arteries other than the middle cerebral artery, where CBv can be captured during related cognitive tasks with different difficulty levels (e.g., n‐back tasks).

## 5 CONCLUSIONS

In conclusion, we validated our first hypothesis: we were able to distinguish between dominant and non‐dominant signals during mental activation generated by ACE‐III cognitive tasks better than with population‐normalized mean peak change analysis by applying an appropriate entropy family, such as dispersion entropy, to brain hemodynamic signals. Then, in response to cognitive stimuli, NVC behavior showed lower statistical complexity measures than baseline but significantly lower SCM values between dominant and non‐dominant hemispheres, depending on the stimulated cognitive function. Additionally, there were no differences in entropic measures between dominant and non‐dominant hemodynamic signals during cognitive effort states.

Finally, our findings suggest that information theory approaches such as entropy and statistical complexity are useful for analyzing the NVC responses to cognitive stimulation, in particular, patterns of brain lateralization.

## FUNDING INFORMATION

This research was funded by ANID National Doctoral Scholarship N° 21221884 and FONDECYT Regular Project N° 1241202. Lucy Beishon is a clinical lecturer funded by the National Institute for Health Research (NIHR). The views expressed in this publication are those of the author(s) and not necessarily those of the NIHR, NHS, or the UK Department of Health and Social Care.

## CONFLICT OF INTEREST STATEMENT

The authors declare that they have no known competing financial interests or personal relationships that could have appeared to influence the work reported in this paper.

## ETHICS STATEMENT

All procedures involving human participants in this study were conducted in accordance with the ethical standards of the institutional and national research committees and with the 1964 Helsinki Declaration and its later amendments (most recently revised in 2024), or comparable ethical standards. Ethical approval for the study protocol was granted by the University of Leicester Research Ethics Committee (Reference No. 5355vjh12cardiovascularsciences). All participants provided written informed consent prior to inclusion in the study, in accordance with approved protocols. No vulnerable populations were involved, and participants were informed of their right to withdraw from the study at any time without consequence.

## Figures and Tables

**FIGURE A2.1:**
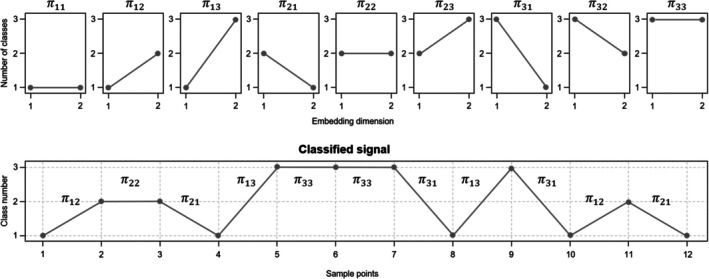
Dispersion patterns with *m* = 2, *c* = 3, and τ = 1. The classified signal corresponds to the univariate signal *x*, described in the example, symbolized as classes.

## Data Availability

The data used in this study is not publicly available due to ethical and privacy considerations related to participant confidentiality. Requests for access to anonymized data may be considered by the Cerebral Hemodynamics in Aging and Stroke Medicine (CHiASM) laboratory upon reasonable request and subject to approval by the University of Leicester ethics committee.

## APPENDIX A

### A1. Mapping techniques used in dispersion entropy

**Linear mapping algorithm**
 *LinearMapping*(x, c){ return *digitize*(x,*arange*(min(x),max(x),*Vpp*(x)/c)); }
where the inputs are x the signal and c the quantity of classes represented by the number of bins. The *digitize* function returns the indices of the bins to which each value in the input array belongs. The *arange* function returns an array corresponding to evenly spaced values within a given interval, whose input parameters are the lowest and highest values of the signal and the difference between them calculated by the $V_{\mathrm{pp}}$ (peak‐to‐peak) function.

**Normal Cumulative Distribution Function (NCDF) algorithm**
 *NCDF*(x, c){ zx = ndtr((x-mean(x))/std(x)); return *digitize*(zx, *arange*(0,1,1/c)); }
where the inputs are x the signal and c the quantity of classes represented by the number of bins. The *ndtr* function is the cumulative distribution of the standard normal distribution:

$$\;\frac{1}{\sigma \sqrt{2\pi }}\int_{-\infty }^{x_{j}}e\frac{-{\left(t-\mu \right)}^{2}}{2\sigma^{2}}\mathrm{dt}$$

And returns the area under the standard Gaussian probability density function. The *digitise* function returns the indices of the bins to which each value in the input array belongs. The *arange* function returns an array corresponding to evenly spaced values within a given interval, whose input parameters are 0, 1, and the equiprobability of classes.

### A2. An example of dispersion entropy calculation

As an example of the above, let's calculate the DE value of x = {61, 68, 66, 64, 72, 71, 73, 63, 74, 62, 65, 60} (*N* = 12) with *m* = 2, *c* = 3, and *τ* = 1. As previously described, first we map x into *c* classes by applying the NCDF algorithm and obtaining $u_{j}$ = {0.12, 0.62, 0.45, 0.29, 0.88, 0.83, 0.91, 0.22, 0.94, 0.16, 0.37, 0.08}. Then, the classified time series $u_{i}^{m,c}$ = {1, 2, 2, 1, 3, 3, 3, 1, 3, 1, 2, 1} is calculated. For this example, there are $N-\left(m-1\right)\tau$ = 12 − (2 − 1)1 = 11 embedding vectors with their $c^{m}=3^{2}$ corresponding dispersion patterns. Considering an embedding dimension of *m* = 2 in this example, the following are the associated dispersion patterns:

$$u_{1}^{2,3}=\left\{1,2\right\}\to \pi_{12},u_{2}^{2,3}=\left\{2,2\right\}\to \pi_{22},u_{3}^{2,3}=\left\{2,1\right\}\to \pi_{21},u_{4}^{2,3}=\left\{1,3\right\}\to \pi_{13},$$

$$u_{5}^{2,3}=\left\{3,3\right\}\to \pi_{33},u_{6}^{2,3}=\left\{3,3\right\}\to \pi_{33},u_{7}^{2,3}=\left\{3,1\right\}\to \pi_{31},u_{8}^{2,3}=\left\{1,3\right\}\to \pi_{13},$$

$$u_{9}^{2,3}=\left\{3,1\right\}\to \pi_{31},u_{10}^{2,3}=\left\{1,2\right\}\to \pi_{12},u_{11}^{2,3}=\left\{2,1\right\}\to \pi_{21}$$

Then, considering the probability of each dispersion pattern the DE value for the univariate signal x is calculated using the Equation (3) as follows: $-\left(\frac{2}{11}\ln\left(\frac{2}{11}\right)+\frac{2}{11}\ln\left(\frac{2}{11}\right)+\frac{2}{11}\ln\left(\frac{2}{11}\right)+\frac{1}{11}\ln\left(\frac{1}{11}\right)+\frac{2}{11}\ln\left(\frac{2}{11}\right)+\frac{2}{11}\ln\left(\frac{2}{11}\right)\right)=1.7678$, and using the equation H_DE_ / ln(c^m^) the normalized DE is equal to 0.8045.

Figure A2.1 represents an example of dispersion patterns with a number of classes equal to three and an embedding dimension of two, as well as the categorized signal and associated patterns.

### A3. Distances and disequilibrium calculations for statistical complexity

Commonly, two complexity approaches are used, Shiner‐Davison‐Landsberg's complexity which measures disorder in a $P_{i}$ through its information measure, and López‐Ruiz et al. (1995) statistical disequilibrium complexity (Q). This last can be applied to any probability space. The disequilibrium $\left(Q\right)$ of a system is obtained from the product of the normalization constant $\left(Q_{0}\right)$ and the distance between the probability distribution associated with the current state of the system $\left(P\right)$ and the uniform probability distribution $\left(\mathrm{Pe}\right)$ of the signal in question as shown in Equation (A1).

(A1)
$$Q=Q_{0}.D\left[P,P_{e}\right]$$

In the case of the Wooters distance ($D_{W}$) is defined by Equation (A2):

(A2)
$$D_{W}\left[P,P_{e}\right]=\cos^{-1}\left\{\sum_{j=1}^{m}{\left(p_{j}^{\left(1\right)}\right)}^{\frac{1}{2}}{\left(p_{j}^{\left(2\right)}\right)}^{\frac{1}{2}}\right\}$$

with the normalization constant $Q_{0}=1/\cos^{-1}\left\{{\left[1/m\right]}^{1/2}\right\}$.

The Shannon Jensen divergence ($D_{S}$) is defined by Equation (A3):

(A3)
$$D_{s}\left[P,P_{e}\right]=Q_{0}\left\{\frac{S\left[P+P_{e}\right]}{2}-\frac{S\left[P\right]}{2}-\frac{S\left[P_{e}\right]}{2}\right\}$$

with the normalization constant $Q_{0}=-2{\left\{\left(\frac{D!+1}{D!}\right)\ln\left[D!+1\right]-2\ln\left[2D!\right]+\ln\left[D!\right]\right\}}^{-1}$.

### A4. The best post hoc test obtained *p*‐values when analyzing CrCP and RAP signals' dispersion entropy (DE) SCM distributions within baseline and each paradigm non‐dominant and dominant hemisphere.

**Table:**

Paradigm	Function	CrCP signals DE SCM			RAP signals DE SCM		
		Baseline/non‐dominant	Baseline/dominant	Non‐dominant/dominant	Baseline/non‐dominant	Baseline/dominant	Non‐dominant/dominant
A1	Attention	**0.001**	0.900	**0.002**	**0.046**	0.901	0.124
A2	Attention	0.504	**0.001**	**0.037**	0.921	**0.015**	**0.043**
A3	Attention	**0.001**	**0.001**	**0.027**	**0.007**	0.760	**0.049**
A4	Attention	0.214	**0.001**	**0.049**	0.792	0.057	**0.010**
A5	Memory	0.109	**0.001**	**0.020**	**0.007**	0.760	**0.049**
A6	Fluency	0.900	**0.014**	**0.027**	**0.000**	**0.000**	**0.048**
A7	Fluency	0.174	0.504	**0.010**	**0.001**	**0.001**	**0.037**
A8	Memory	**0.001**	0.504	**0.014**	0.109	**0.001**	**0.020**
A9	Memory	**0.001**	0.261	0.065	0.261	0.632	**0.037**
B1	Language	0.504	**0.003**	0.085	0.900	0.085	0.138
B2	Language	**0.000**	0.287	**0.012**	0.900	**0.049**	**0.049**
B3	Language	0.888	**0.037**	**0.010**	0.824	0.261	0.085
B4	Language	0.900	**0.020**	**0.020**	**0.001**	**0.027**	**0.027**
B5	Language	0.109	0.900	**0.049**	0.760	0.373	0.109
B6	Language	0.900	**0.049**	**0.020**	**0.001**	0.065	0.065
C1	Visuospatial	0.373	0.373	**0.020**	0.900	**0.010**	**0.014**
C2	Visuospatial	**0.002**	0.097	0.315	0.109	0.900	**0.049**
C3	Visuospatial	0.760	0.214	**0.049**	**0.001**	0.900	**0.002**
C4	Visuospatial	**0.005**	**0.001**	**0.037**	**0.001**	**0.001**	**0.020**
C5	Memory	**0.001**	**0.001**	0.174	**0.001**	**0.001**	0.109

*Note*: All statistical tests had a significance level of 0.05, *p*‐values lower than the significance level are highlighted in bold font.

### A5. Mean ROC AUC values for different CBv signal entropy and SCM classifications in sections A, B, and C.

**Table:**

Section	Baseline/non‐dominant	Baseline/dominant	Non‐dominant/dominant
A	**0.707**	**0.761**	0.423
B	0.602	0.627	0.543
C	**0.704**	0.634	0.546

*Note*: Acceptable ROC AUC scores (≥0.70) are highlighted in bold font.

## References

[phy270492-bib-0001] Aaslid, R. (1987). Visually evoked dynamic blood flow response of the human cerebral circulation. Stroke, 18(4), 771–775. 10.1161/01.str.18.4.771 3299883

[phy270492-bib-0002] Aslin, R. N. , Shukla, M. , & Emberson, L. L. (2015). Hemodynamic correlates of cognition in human infants. Annual Review of Psychology, 66(1), 349–379. 10.1146/annurev-psych-010213-115108 PMC4429889 25251480

[phy270492-bib-0003] Azami, H. , & Escudero, J. (2018). Amplitude‐ and fluctuation‐based dispersion entropy. Entropy, 20(3), 210. 10.3390/e20030210 33265301 PMC7512725

[phy270492-bib-0004] Azami, H. , Rostaghi, M. , Abasolo, D. , & Escudero, J. (2017). Refined composite multiscale dispersion entropy and its application to biomedical signals. IEEE Transactions on Biomedical Engineering, 64(12), 2872–2879. 10.1109/tbme.2017.2679136 28287954

[phy270492-bib-0005] Azevedo, E. , Santos, R. , Freitas, J. , Rosas, M.‐J. , Gago, M. , Garrett, C. , & Rosengarten, B. (2010). Deep brain stimulation does not change neurovascular coupling in non‐motor visual cortex: An autonomic and visual evoked blood flow velocity response study. Parkinsonism and Related Disorders, 16(9), 600–603. 10.1016/j.parkreldis.2010.08.016 20846894

[phy270492-bib-0007] Beishon, L. , Williams, C. A. L. , Panerai, R. B. , Robinson, T. G. , & Haunton, V. J. (2017). Reproducibility of task activation using the Addenbrooke's cognitive examination in healthy controls: A functional transcranial Doppler ultrasonography study. Journal of Neuroscience Methods, 291, 131–140. 10.1016/j.jneumeth.2017.08.019 28827165

[phy270492-bib-0008] Beishon, L. , Williams, C. A. L. , Robinson, T. G. , Haunton, V. J. , & Panerai, R. B. (2018). Neurovascular coupling response to cognitive examination in healthy controls: A multivariate analysis. Physiological Reports, 6(14), e13803. 10.14814/phy2.13803 30033685 PMC6055030

[phy270492-bib-0009] Beishon, L. C. , Intharakham, K. , Haunton, V. J. , Robinson, T. G. , & Panerai, R. B. (2021). The interaction of dynamic cerebral autoregulation and neurovascular coupling in cognitive impairment. Current Alzheimer Research, 18(14), 1067–1076. 10.2174/1567205019666211227102936 35026972

[phy270492-bib-0010] Beishon, L. C. , Williams, C. A. L. , Panerai, R. B. , Robinson, T. G. , & Haunton, V. J. (2018). The assessment of neurovascular coupling with the Addenbrooke's cognitive examination: A functional transcranial Doppler ultrasonographic study. Journal of Neurophysiology, 119(3), 1084–1094. 10.1152/jn.00698.2017 29187557

[phy270492-bib-0013] Castro, P. , Santos, R. , Freitas, J. , Rosengarten, B. , Panerai, R. , & Azevedo, E. (2012). Adaptation of cerebral pressure‐velocity hemodynamic changes of neurovascular coupling to orthostatic challenge. Perspectives in Medicine, 1(1–12), 290–296. 10.1016/j.permed.2012.02.052

[phy270492-bib-0015] Deppe, M. , Ringelstein, E. B. , & Knecht, S. (2004). The investigation of functional brain lateralization by transcranial Doppler sonography. NeuroImage, 21(3), 1124–1146. 10.1016/j.neuroimage.2003.10.016 15006680

[phy270492-bib-0016] Diykh, M. , Abdulla, S. , Deo, R. C. , Siuly, S. , & Ali, M. (2023). Developing a novel hybrid method based on dispersion entropy and adaptive boosting algorithm for human activity recognition. Computer Methods and Programs in Biomedicine, 229, 107305. 10.1016/j.cmpb.2022.107305 36527814

[phy270492-bib-0017] Evans, D. H. , Levene, M. I. , Shortland, D. B. , & Archer, L. N. J. (1988). Resistance index, blood flow velocity, and resistance‐area product in the cerebral arteries of very low birth weight infants during the first week of life. Ultrasound in Medicine & Biology, 14(2), 103–110. 10.1016/0301-5629(88)90176-7 3279689

[phy270492-bib-0018] Flood, M. W. , & Grimm, B. (2021). EntropyHub: An open‐source toolkit for entropic time series analysis. PLoS One, 16(11), e0259448. 10.1371/journal.pone.0259448 34735497 PMC8568273

[phy270492-bib-0020] Girouard, H. , & Iadecola, C. (2006). Neurovascular coupling in the normal brain and in hypertension, stroke, and Alzheimer disease. Journal of Applied Physiology, 100(1), 328–335. 10.1152/japplphysiol.00966.2005 16357086

[phy270492-bib-0022] Güntürkün, O. , & Ocklenburg, S. (2017). Ontogenesis of lateralisation. Neuron, 94(2), 249–263. 10.1016/j.neuron.2017.02.045 28426959

[phy270492-bib-1001] Hosford, P. S. , & Gourine, A. V. (2019). What is the key mediator of the neurovascular coupling response? Neuroscience & Biobehavioral Reviews, 96, 174–181. 10.1016/j.neubiorev.2018.11.011 30481531 PMC6331662

[phy270492-bib-0024] Hsieh, S. , Schubert, S. , Hoon, C. , Mioshi, E. , & Hodges, J. R. (2013). Validation of the Addenbrooke's cognitive examination III in frontotemporal dementia and Alzheimer's disease. Dementia and Geriatric Cognitive Disorders, 36(3–4), 242–250. 10.1159/000351671 23949210

[phy270492-bib-0025] Iadecola, C. (2017). The neurovascular unit coming of age: A journey through neurovascular coupling in health and disease. Neuron, 96(1), 17–42. 10.1016/j.neuron.2017.07.030 28957666 PMC5657612

[phy270492-bib-0026] Kolmogorov, A. N. (1958). A new metric invariant of transient dynamical systems and automorphisms in Lebesgue spaces. Doklady Akademii Nauk SSSR, 119, 861–864.

[phy270492-bib-0027] Kowalski, A. M. , Martín, M. T. , Plastino, A. , Rosso, O. A. , & Casas, M. (2011). Distances in probability space and the statistical complexity setup. Entropy, 13(6), 1055–1075. 10.3390/e13061055

[phy270492-bib-1002] Leacy, J. K. , Johnson, E. M. , Lavoie, L. R. , Macilwraith, D. N. , Bambury, M. , Martin, J. A. , Lucking, E. F. , Linares, A. M. , Saran, G. , Sheehan, D. P. , Sharma, N. , Day, T. A. , & O'Halloran, K. D. (2022). Variation within the visually evoked neurovascular coupling response of the posterior cerebral artery is not influenced by age or sex. Journal of Applied Physiology, 133(2), 335–348. 35771218 10.1152/japplphysiol.00292.2021 PMC9359642

[phy270492-bib-0028] Letzner, S. , Güntürkün, O. , Lor, S. , Pawlik, R. J. , & Manns, M. (2017). Visuospatial attention in the lateralised brain of pigeons—A matter of ontogenetic light experiences. Scientific Reports, 7(1), 15547. 10.1038/s41598-017-15796-6 29138476 PMC5686156

[phy270492-bib-0029] López‐Ruiz, R. , Mancini, H. L. , & Calbet, X. (1995). A statistical measure of complexity. Physics Letters A, 209(5–6), 321–326. 10.1016/0375-9601(95)00867-5

[phy270492-bib-0030] Malatesta, G. , & Tommasi, L. (2023). Editorial: Expert opinion in environmental and genetic factors impacting functional brain lateralization in development and evolution. Frontiers in Behavioral Neuroscience, 17, 1215176. 10.3389/fnbeh.2023.1215176 37324522 PMC10264780

[phy270492-bib-0032] Mioshi, E. , Dawson, K. , Mitchell, J. , Arnold, R. , & Hodges, J. R. (2006). Addenbrooke's Cognitive Examination—Revised [dataset]. In PsycTESTS Dataset. American Psychological Association (APA). 10.1037/t05533-000

[phy270492-bib-0033] Moody, M. , Panerai, R. B. , Eames, P. J. , & Potter, J. F. (2005). Cerebral and systemic hemodynamic changes during cognitive and motor activation paradigms. American Journal of Physiology. Regulatory, Integrative and Comparative Physiology, 288(6), R1581–R1588. 10.1152/ajpregu.00837.2004 15677522

[phy270492-bib-0034] Panerai, R. B. , Haunton, V. J. , Llwyd, O. , Minhas, J. S. , Katsogridakis, E. , Salinet, A. S. , Maggio, P. , & Robinson, T. G. (2021). Cerebral critical closing pressure and resistance‐area product: The influence of dynamic cerebral autoregulation, age and sex. Journal of Cerebral Blood Flow & Metabolism, 41(9), 2456–2469. 10.1177/0271678x211004131 33818187 PMC8392773

[phy270492-bib-1003] Rosengarten, B. , Molnar, S. , Trautmann, J. , & Kaps, M. (2006). Simultaneous VEP and transcranial Doppler ultrasound recordings to investigate activation‐flow coupling in humans. Ultrasound in Medicine & Biology, 32(8), 1171–1180. 16875952 10.1016/j.ultrasmedbio.2006.04.016

[phy270492-bib-0037] Rostaghi, M. , & Azami, H. (2016). Dispersion entropy: A measure for time‐series analysis. IEEE Signal Processing Letters, 23(5), 610–614. 10.1109/lsp.2016.2542881

[phy270492-bib-0038] Salinet, A. S. , Silva, N. C. , Caldas, J. , de Azevedo, D. S. , de‐Lima‐Oliveira, M. , Nogueira, R. C. , Conforto, A. B. , Texeira, M. J. , Robinson, T. G. , Panerai, R. B. , & Bor‐Seng‐Shu, E. (2018). Impaired cerebral autoregulation and neurovascular coupling in middle cerebral artery stroke: Influence of severity? Journal of Cerebral Blood Flow & Metabolism, 39(11), 2277–2285. 10.1177/0271678x18794835 30117360 PMC6827118

[phy270492-bib-0039] Shannon, C. E. (1948). A mathematical theory of communication. Bell System Technical Journal, 27(4), 623–656. 10.1002/j.1538-7305.1948.tb00917.x

[phy270492-bib-0040] Sinai, J. (1959). On the concept of entropy for a dynamic system. Doklady Akademii Nauk SSSR, 124, 768–771.

[phy270492-bib-0041] Stroobant, N. , & Vingerhoets, G. (2000). Transcranial Doppler ultrasonography monitoring of cerebral haemodynamics during performance of cognitive tasks: A review. Neuropsychology Review, 10, 213–231. 10.1023/a:1026412811036 11132101

[phy270492-bib-0042] Telesford, Q. K. , Simpson, S. L. , Burdette, J. H. , Hayasaka, S. , & Laurienti, P. J. (2011). The brain as a complex system: Using network science as a tool for understanding the brain. Brain Connectivity, 1(4), 295–308. 10.1089/brain.2011.0055 22432419 PMC3621511

[phy270492-bib-0043] Wang, L. , Du, T. , Zhao, L. , Shi, Y. , & Zeng, W. (2023). Research on the lateralization of brain functional complexity in mild cognitive impairment‐Alzheimer's disease progression based on multiscale lateralized brain entropy. Biomedical Signal Processing and Control, 86, 105216. 10.1016/j.bspc.2023.105216

[phy270492-bib-0045] Woodhead, Z. V. J. , Rutherford, H. A. , & Bishop, D. V. M. (2020). Measurement of language laterality using functional transcranial Doppler ultrasound: A comparison of different tasks. Wellcome Open Research, 3, 104. 10.12688/wellcomeopenres.14720.3 30345386 PMC6171558

[phy270492-bib-0046] Zanin, M. , Zunino, L. , Rosso, O. A. , & Papo, D. (2012). Permutation entropy and its Main biomedical and econophysics applications: A review. Entropy, 14(8), 1553–1577. 10.3390/e14081553

[phy270492-bib-0047] Zhang, T. , Huang, W. , Wu, X. , Sun, W. , Lin, F. , Sun, H. , & Li, J. (2021). Altered complexity in resting‐state fNIRS signal in autism: A multiscale entropy approach. Physiological Measurement, 42(8), 085004. 10.1088/1361-6579/ac184d 34315139

